# Reliability and Practicability of FAP-RADS Version 1.0 for Structured Reporting of FAPI Imaging in Pulmonary and Urothelial Carcinoma

**DOI:** 10.2967/jnumed.125.271080

**Published:** 2026-05

**Authors:** Emil Novruzov, Eduards Mamlins, Liam Widjaja, Sophie C. Siegmund, Tadashi Watabe, Yuriko Mori, Christian Stief, Amanda Tufman, Ken Herrmann, Michael A. Gorin, Martin G. Pomper, Steven P. Rowe, Frederik L. Giesel, Rudolf A. Werner, Gabriel T. Sheikh

**Affiliations:** 1 Department of Nuclear Medicine, Medical Faculty and University Hospital Duesseldorf, Heinrich-Heine-University Duesseldorf, Düsseldorf, Germany;; 2 Department of Nuclear Medicine, LMU University Hospital, Munich, Germany;; 3 Bavarian Cancer Research Center, Munich, Germany;; 4 Department of Radiology, Osaka University Graduate School of Medicine, Osaka, Japan;; 5 Department of Urology, LMU University Hospital, Munich, Germany;; 6 Department of Medicine V, LMU University Hospital, Munich, Germany;; 7 Department of Nuclear Medicine, University of Duisburg-Essen, Essen, Germany;; 8 Milton and Carroll Petrie Department of Urology, Icahn School of Medicine at Mount Sinai, New York, New York;; 9 Department of Radiology, University of Texas Southwestern Medical Center, Dallas, Texas;; 10 Institute for Radiation Sciences, Osaka University, Osaka, Japan;; 11 Center for Integrated Oncology Aachen Bonn Cologne Düsseldorf, Düsseldorf, Germany; and; 12 Russell H. Morgan Department of Radiology and Radiological Science, Division of Nuclear Medicine and Molecular Imaging, Johns Hopkins School of Medicine, Baltimore, Maryland

**Keywords:** FAP-RADS, FAP PET, FAP imaging, standardized reporting, RADS framework

## Abstract

Recently, a collaborative effort among research centers in the United States and Europe resulted in the development of fibroblast activation protein reporting and data system (FAP-RADS) version 1.0, a reporting framework for fibroblast activation protein (FAP)–targeted imaging using the fundamental principles of the molecular imaging reporting and data systems framework. This retrospective study aimed to investigate the reliability and consistency of FAP-RADS version 1.0 framework for patients with lung cancer and urothelial cancer. **Methods:** Four readers with varying levels of PET/CT experience, working without access to clinical patient information (2 readers with more than 10 y of experience, 1 reader with 6 y of experience, and a nuclear medicine resident with less than 3 y of experience) evaluated PET/CT scans from a total of 60 patients (30 with lung cancer and 30 with urothelial cancer). Readers were instructed to identify no more than 5 target lesions (TLs) per patient, with a maximum of 3 TLs per organ or compartment. Each evaluation required the assignment of a final overall FAP-RADS score, defined as the highest score among the assessed TLs. **Results:** Interreader agreement for the overall scan impression and the presence of lymph node metastases was moderate to substantial, with κ values of 0.62 (95% CI, 0.52–0.73) and 0.59 (95% CI, 0.49–0.69), respectively. Interreader agreement regarding referral for radiopharmaceutical therapy on the basis of tracer uptake intensity and disease extent were high, with κ values of 0.70 (95% CI, 0.60–0.81) and 0.66 (95% CI, 0.55–0.76), respectively. The internal consistency for TL-based assessments and overall FAP-RADS score was almost perfect, with a Cronbach α of 0.92 and 0.88, respectively. Analyses of TL-based FAP-RADS scores demonstrated high interreader agreement for both experienced and less experienced readers, with weighted κ values of 0.72 (95% CI, 0.53–0.91) and 0.75 (95% CI, 0.57–0.92), respectively. **Conclusion:** The FAP-RADS version 1.0 framework appears to be a robust tool for standardized reporting in both clinical studies and routine clinical practice, demonstrating substantial interreader agreement across most evaluated categories. Further studies are warranted to validate its applicability in additional tumor entities and with other FAP-targeting ligands.

## Visual Abstract

**Figure:**
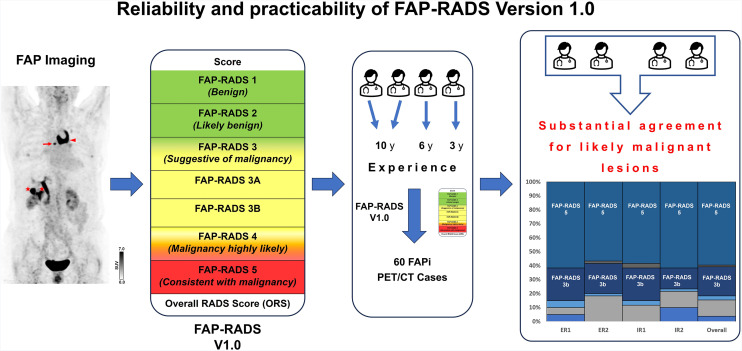

Fibroblast activation protein (FAP) imaging has been demonstrated to be a highly promising diagnostic approach because of the upregulation of its expression in approximately 90% of epithelial malignancies. Certain benign conditions, such as chronic inflammatory or fibrotic processes, also display FAP upregulation because of the activation of cancer-associated fibroblasts (1).

Accurate estimation of tumor extent before therapy initiation and monitoring therapeutic response are crucial steps for optimal, personalized therapeutic decision-making. Given the pancancer feature and incidental benign findings on FAP-targeted imaging, there was an unmet clinical need for a unified clinical and research language to enable better comparability of results across research centers and streamline the reporting process in clinical contexts. Recently, a collaborative effort among research centers in the United States and Europe resulted in the development of FAP reporting and data system (FAP-RADS) version 1.0, a reporting framework for FAP-targeted imaging based on the molecular imaging RADS (MI-RADS), analogous to frameworks for imaging prostate-specific membrane antigen (PSMA-RADS) and somatostatin receptors (SSRT-RADS). Like other MI-RADS frameworks, FAP-RADS version 1.0 is intended to promote acceptance and comprehensive applicability among molecular imaging experts in both research and clinical settings (2–6).

The main goals of FAP-RADS version 1.0 were to streamline the reporting process, improve reliability in the research setting, and facilitate communication among referring specialists from different disciplines to guide the initiation of further work-up (e.g., for equivocal findings) or therapeutic decision-making, including radiopharmaceutical therapy (RPT). Moreover, FAP-RADS version 1.0 is a semiqualitative assessment system, mandating the consideration of both disease- and radiotracer-specific details to minimize the frequency of misinterpretation and better stratification of imaging pitfalls. Consistent with other RADS frameworks, FAP-RADS version 1.0 assigns a score to each lesion of interest, using a 1–5 Likert-type scale (1 = definitively benign; 5 = consistent with malignancy), and provides an overall impression of the imaging study to communicate the level of certainty to the referring physician (Supplemental Table 1, available at http://jnm.snmjournals.org) (2–6).

Given the broad applicability of FAP-targeted imaging across numerous epithelial malignancies, the reliability of FAP-RADS version 1.0 must be validated across various malignancies and reader experience levels. Moreover, FAP-RADS version 1.0 necessitates the consideration of clinical context and specific features of the diagnosis from the referring physician. Current research has used FAP inhibitors (FAPIs) as FAP ligands, which have demonstrated particularly remarkable results for gastrointestinal tumors, lung cancer (LC), urothelial cancer (UC), and ovarian cancer when compared with conventional imaging (7–13).

We sought to assess the interreader reliability of FAP-RADS version 1.0 in patients with LC and UC by readers with varying levels of experience who were masked to the patients’ medical history.

## MATERIALS AND METHODS

### Patient Population

We retrospectively evaluated 60 patients (15 women, 45 men) with biopsy-proven LC or UC who underwent FAPI imaging for primary staging between May 2021 and April 2025. Table 1 depicts the baseline characteristics of the enrolled patients. The study was approved by the institutional review board of Ludwig-Maximilians-University Munich (project 25-0229) and conducted in accordance with the ethical standards of the institutional or national research committees and the 1964 Declaration of Helsinki and its later amendments or comparable ethical standards.

**TABLE 1.** Patient Characteristics

Characteristic	LC (*n* = 30)	UC (*n* = 30)
Age (y)	68 ± 8	51 ± 11
Sex		
Male	19	26
Female	11	4
Indication for FAPI PET/CT		
Primary staging	30	30

### Image Acquisition and Analysis

Whole-body and total-body PET/CT scans, with or without contrast, were performed for all patients approximately 60 min after intravenous injection of [^18^F]AlF-FAPI-74. The median injected activity overall was 203 MBq (range, 110–295 MBq), with 201 MBq (range, 110–295 MBq) in patients with LC and 206 MBq (range, 110–291 MBq) in those with UC. Nine patients in the UC subgroup received [^68^Ga]Ga-FAPI-46 (median activity, 191 MBq; range, 110–252 MBq) on the same hybrid PET/CT scanner (Biograph mCT Flow 20; Siemens Healthineers). Supplemental Table 2 summarizes the scan protocol. All acquired PET/CT scans were analyzed using a dedicated software package (Visage 7.1; Pro Medicus Ltd.). SUVs were determined by drawing a region of interest on target lesions (TLs) on the transaxial plane, which were automatically converted to a 3-dimensional volume of interest using a 40% isocontouring approach.

### Reading

The PET/CT scans of all 60 patients (1 per person) were evaluated by 4 readers; 3 board-certified nuclear medicine experts (2 readers with >10 y of experience and 1 reader with 6 y of experience) and 1 nuclear medicine resident (<3 y of experience in PET/CT imaging). Readers were masked to all patient data except age, sex, and referral diagnosis. All readers were familiar with the workstations and software from clinical practice and were trained for correct interpretation of recently published FAP-RADS version 1.0 (2). Consistent with the prerequisites of the framework, readers were limited to selecting no more than 5 TLs per patient and up to 3 TLs per organ or compartment. Each reading required a final overall FAP-RADS score, defined as the highest score among the assessed TLs. Although FAP-RADS version 1.0 encourages the selection of dominant lesions on the basis of morphologic size or tracer uptake, the selection was ultimately left to the discretion of the readers.

Various features of TLs were assessed by readers, such as overall impression of the scan in terms of presence of malignancy and FAPI uptake, organ and lymph node metastases (LNM), location, SUV_max_, specific FAP-RADS scoring of each TL, and eligibility for eventual FAP-targeted RPT on the basis of both the assigned scores and the general image impression. The concordance for the overall FAP-RADS score between experienced and inexperienced readers was also evaluated.

### Statistical Analysis

Clinical and demographic characteristics are presented using descriptive statistics. Categoric data are displayed as number and frequency; continuous data are displayed as mean ± SD. A *P* value of less than 0.05 was considered statistically significant. Interreader agreement was measured by calculating κ statistics. Pairwise interreader agreement for ordinal parameters was conducted by weighted κ test. κ scale was interpreted as follows: poor agreement (0), slight agreement (0.01–0.20), fair agreement (0.21–0.40), moderate agreement (0.41–0.60), substantial agreement (0.61–0.80), and almost-perfect agreement (0.81–1.00). In the course of statistical assessment, FAP-RADS scores of 1 and 2 were merged, as they are unlikely to have any clinical impact.

Interreader and intrareader agreement of multiple readers (>2 readers) for continuous parameters was calculated using the intraclass correlation coefficient (ICC) and 95% CI. According to Koo and Li, agreement is considered poor for an ICC of less than 0.5, moderate for an ICC of 0.5–0.75, good for an ICC of 0.75–0.90, and excellent for an ICC greater than 0.90 (14). Cronbach α was calculated as a measure of internal consistency. To assess interreader agreement for ordinal data, the Kendall coefficient of concordance (*W*) was calculated and interpreted as poor (0.10–0.30), slight (0.31–0.50), moderate (0.51–0.70), substantial (0.71–0.90), or almost perfect (0.91–1.00).

Statistical analyses were performed using Excel version 2311 (Microsoft Corp.), DataTab (now Numiqo) statistics calculator (https://numiqo.com), and SigmaPlot 11 (Systat Software). In this study, data cleansing was performed to identify and correct any errors, inconsistencies, or missing values in the dataset, thus improving the overall quality and reliability of the data.

## RESULTS

### Interreader Agreement for Parameters with a Binary Outcome

**FIGURE 1.**
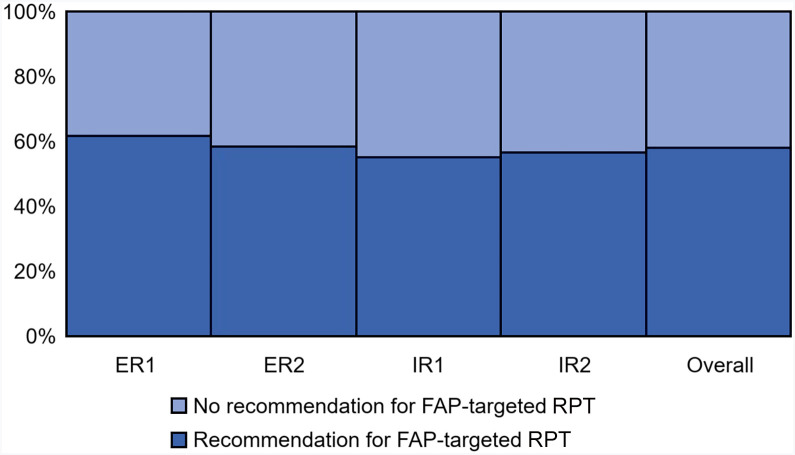
Recommendation for FAP-targeted RPT. ER = experienced reader; IR = inexperienced reader.

Four readers assessed the parameters with a binary outcome—overall scan impression, LNM, and referral rate for RPT—in the total cohort and in the UC and LC subgroups (Table 2). Accordingly, the overall scan impression and LNM in the total cohort revealed a moderate to substantial interreader agreement, with a κ of 0.62 (95% CI, 0.52–0.73) and 0.59 (95% CI, 0.49–0.69), respectively. The readers were also asked to refer the patients for RPT using 2 strategies (i.e., tracer uptake intensity and disease extent), although we acknowledge the lack of clearly defined clinical guidelines for this topic. Thus, the readers had to interpret the findings using their previous experience with other comparable theranostic platforms and clinical intuition. Consistent with the results for overall scan impression, referral for RPT on the basis of FAPI uptake intensity and disease extent showed good interreader agreement, with a κ of 0.70 (95% CI, 0.60–0.81) and 0.66 (95% CI, 0.55–0.76), respectively (Fig. 1).

**TABLE 2.** Interreader Agreement for Parameters with Binary Outcome

Parameter	All patients	LC	UC
Overall scan impression	0.62 (0.52–0.73)	0.32 (0.17–0.47)	0.68 (0.54–0.82)
Lymph node involvement	0.59 (0.49–0.69)	0.41 (0.26–0.55)	0.77 (0.62–0.91)
RPT referral			
Uptake intensity	0.70 (0.60–0.81)	0.67 (0.53–0.81)	0.68 (0.54–0.83)
Disease extent	0.66 (0.55–0.76)	0.53 (0.38–0.68)	0.76 (0.61–0.91)

Data expressed as κ value, followed by 95% CI in parentheses.

Interreader agreement differed notably between the LC and UC subgroups. In the LC group, interreader agreement was lower for overall scan impression (κ = 0.32; 95% CI, 0.17–0.47) and LNM (κ = 0.41; 95% CI, 0.26–0.55). The UC cohort displayed better results, with a κ of 0.68 (95% CI, 0.54–0.82) for overall scan impression and 0.77 (95% CI, 0.62–0.91) for LNM. The referral rates for RPT based on FAPI uptake intensity and disease extent were substantial for both subgroups. The κ values for referral rates for uptake intensity and disease extent were 0.67 (95% CI, 0.53–0.81) and 0.53 (95% CI, 0.38–0.68), respectively, in the LC subgroup and 0.68 (95% CI, 0.54–0.83) and 0.76 (95% CI, 0.61–0.91), respectively, in the UC subgroup.

### Interreader Agreement for FAP-RADS and Other Ordinal Parameters

Before proceeding with interreader agreement for FAP-RADS version 1.0 and other ordinal parameters, we assessed the consistency of the key prerequisites of FAP-RADS version 1.0. Effective use of FAP-RADS version 1.0 requires low interreader variability in tracer uptake measurements for both background and TLs. SUV_max_ measurements in the descending aorta demonstrated excellent internal consistency, with a Cronbach α of 0.97 among all 4 readers. Moreover, interreader agreement for background SUV_max_ was almost perfect, with an ICC of 0.89 (95% CI, 0.84–0.92). Internal consistency for qualitative assessment of FAPI uptake was also excellent, with a Cronbach α of 0.91.

**FIGURE 2.**
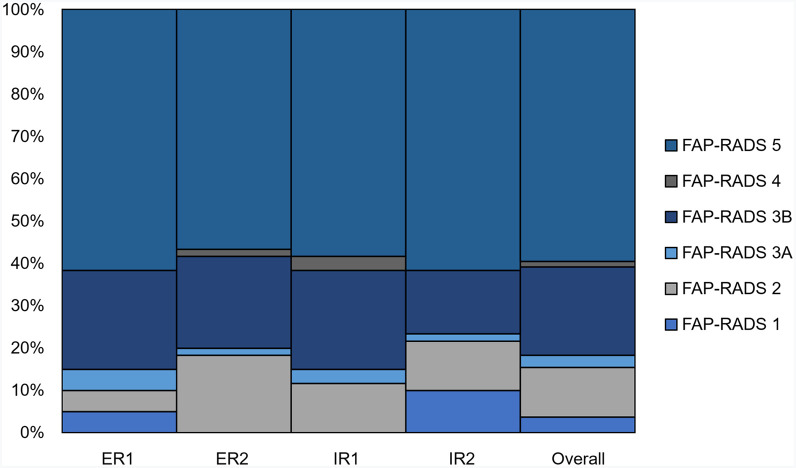
Overview of distribution of overall FAP-RADS score among all readers for total cohort of 60 patients. There was substantial agreement among all readers, especially for likely malignant lesions with FAP-RADS score of ≥3. ER = experienced reader; IR = inexperienced reader.

In addition to accurate stratification of TLs, the final assessment by molecular imaging experts remains the most important part of reporting for referring physicians in routine clinical practice. FAP-RADS version 1.0 was designed to support this process with the overall FAP-RADS score (2,3). Figure 2 depicts the distribution of overall FAP-RADS scores among readers. The internal consistency of FAP-RADS version 1.0 in defining the overall FAP-RADS score appeared to be almost perfect, with a Cronbach α of 0.88. The interreader agreement among all 4 readers was also good (Kendall *W* = 0.66). Interreader agreement was substantial in the LC group (Kendall *W* = 0.69) and good in the UC group (Kendall *W* = 0.57).

**FIGURE 3.**
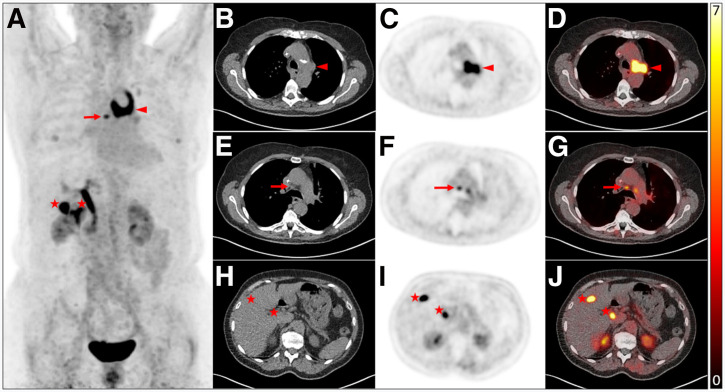
[^18^F]AlF-FAPI-74 PET/CT of 72-y-old woman undergoing primary staging for LC. (A) Maximum-intensity projection image shows intense uptake in central/hilar mass and precarinal lymph node (red arrows), alongside physiologic uptake in the gallbladder, biliary tract (asterisks), kidneys, and bladder. Central mass was rated FAP-RADS 5 by all readers attributable to intense uptake and typical CT morphology (B–D). (E–G) Precarinal lymph node was classified as FAP-RADS 3B by experienced readers; inexperienced readers rated it as FAP-RADS 5 and 3A because of uptake near threshold and minor PET/CT mismatch, respectively. (H–J) Benign uptake in biliary tract and urinary system was consistently rated FAP-RADS 1.

Accurate identification of LNM in epithelial malignancies is quite relevant for correct staging and therapy planning. In this regard, the internal consistency for LNM among all 4 readers was perfect, with a Cronbach α of 0.95. The interreader agreement among all 4 readers showed a substantial agreement (Kendall *W* = 0.83) (Fig. 3).

We also assessed the consistency and interreader agreement of FAP-RADS version 1.0 for TL-based reporting. For TLs detected by all 4 readers, the lesion-based internal consistency of FAP-RADS version 1.0 was excellent, with a Cronbach α of 0.92 in the total cohort. The interreader agreement revealed substantial agreement among all readers (Kendall *W* = 0.76). In the LC subgroup, Cronbach α and Kendall *W* were 0.92 and 0.80, respectively. For the UC subgroup, our analysis indicated good internal consistency and interreader agreement of FAP-RADS version 1.0, with a Cronbach α of 0.82 and Kendall *W* of 0.59, respectively.

The experience level of imaging experts in regular clinical care may be an important variable, given the heterogeneity of the FAPI uptake pattern. Thus, we analyzed its effect on parameters important for clinical decision-making. The recommendation for RPT on the basis of FAPI uptake intensity had almost-perfect interreader agreement among experienced readers (κ = 0.83), whereas interreader agreement among inexperienced readers was substantial (κ = 0.70). The recommendation for RPT on the basis of disease extent also demonstrated substantial interreader agreement between experienced (κ = 0.64) and inexperienced (κ = 0.70) readers. For the overall FAP-RADS score, interreader agreement was substantially high among experienced readers (κ = 0.69), but only moderate among inexperienced readers (κ = 0.52). For TL-based FAP-RADS scores, interreader agreement among experienced and inexperienced readers was high, with weighted κ values of 0.72 (95% CI, 0.53–0.91) and 0.75 (95% CI, 0.57–0.92), respectively.

## DISCUSSION

Since the introduction of the first clinically viable FAP-targeting radiopharmaceuticals, the use of FAPI imaging has rapidly expanded across oncology in both clinical and research settings. This necessitated a reliable reporting system to enable thorough data analysis and comparability across centers, leading to the development of FAP-RADS version 1.0 as a more systematic approach for interpreting FAP-targeted imaging (2,15–19). As FAP-RADS version 1.0 is intended to facilitate communication between imaging experts and referring clinicians, support clinical trial analyses, and reinforce the use of artificial intelligence in FAPI imaging, this study investigated the interreader agreement and reliability of this reporting framework using a bicentric, retrospective design.

We investigated the reliability of FAP-RADS version 1.0 using 2 complementary approaches at both scan- and lesion-based levels, while considering the influence of reader experience. These included visual assessments with binary outcomes (e.g., overall scan impression, eligibility for RPT), and structured Likert-type ordinal evaluations (e.g., overall FAP-RADS scores, FAP-RADS assignment).

Visual assessment of the cohort—including overall scan impression, LNM, and virtual referral rate on the basis of tracer uptake intensity and disease extent—revealed good interreader agreement among all readers. Accurate assessment of predefined lymph node regions is crucial for properly staging epithelial malignancies. The subgroup analysis revealed lesser interreader agreement for the LC subgroup compared with the UC group for both overall scan impression and LNM. In this regard, the visual assessment of lymph node regions in the LC subgroup revealed only moderate interreader agreement (κ = 0.41).

When considering the target lymph nodes assigned an FAP-RADS score by all readers, interreader agreement was excellent (Kendall *W* = 0.83), accompanied by perfect internal consistency for FAP-RADS version 1.0 (Cronbach α = 0.95). FAP-RADS version 1.0 relies on qualitative interpretation of data informed by prior medical history and nonspecific confounding findings. However, this information was intentionally withheld from readers in the present study. Given the high prevalence of concordant benign processes, LC is especially prone to such variability. Therefore, despite the overall good performance of the reporting system, the study design may have contributed to lower interreader agreement rate for certain aspects in the LC subgroup, such as lung lymph node stratification. This impression is supported by the substantial interreader agreement observed for lymph node stratification in the UC group (κ = 0.77). The current evidence indicates a high specificity of FAPI imaging for lymph node stratification, suggesting that interreader agreement may be higher in a real-world setting or study design that does not mask readers to patients’ medical history (20).

Internal consistency of FAP-RADS version 1.0 regarding the estimation of FAPI uptake intensity and measurement of background FAPI uptake in the descending aorta was excellent, with an ICC exceeding 0.90. The virtual referral rates for RPT in both the LC and UC subgroups were comparable. No significant difference in RPT referral rates on the basis of disease extent were observed between experienced and inexperienced readers, although interreader agreement was almost perfect among experienced readers with respect to RPT referral rates on the basis of tracer uptake intensity (Cohen κ = 0.83) (Tables 2 and 3). We consider this finding highly plausible within current decision-making for theranostics. Given the established importance of tracer uptake intensity of TLs for RPT success, experienced readers tend to rely on this feature. However, ongoing research of FAP-targeted RPT has been restricted to small compassionate-use cohorts, and widely accepted algorithms for use in routine clinical care are lacking (19,21–23).

**TABLE 3.** Interreader Agreement for Ordinally Scaled FAP-RADS Version 1.0 Parameters

Parameter	Internal consistency*	Interreader agreement
SUV_max_ in descending aorta		
Total cohort	0.97	0.89^†^
FAPI uptake intensity (qualitative assessment)		
Total cohort	0.91	—
Overall FAP-RADS score		
Total cohort	0.88	0.66^†^
LC	0.88	0.69^‡^
UC	0.88	0.57^‡^
Lymph node involvement		
Total cohort	0.95	0.83^‡^
TL reporting		
Total cohort	0.92	0.76^‡^
LC	0.92	0.80^‡^
UC	0.82	0.59^‡^
RPT referral based on FAPI uptake intensity		
Experienced reader	—	0.83^§^
Inexperienced reader	—	0.70^§^
RPT referral based on disease extent		
Experienced reader	—	0.64^§^
Inexperienced reader	—	0.70^§^
Overall FAP-RADS score		
Experienced reader	—	0.69^§^
Inexperienced reader	—	0.52^§^
TL-based FAP-RADS score		
Experienced reader	—	0.72^§^
Inexperienced reader	—	0.75^§^

* Data expressed as Cronbach α.

† Data expressed as ICC.

‡ Data expressed as Kendall *W*.

§ Data expressed as κ.

The main limitations of this study were the lack of histologic validation of TLs and the masking of readers to patients’ medical history. In accordance with the principles of FAP-RADS version 1.0, false-positive findings would not interfere with the integrity of the study results, as our primary goal was the reproducibility of FAP-RADS scores rather than their predictive value for TLs. Not providing readers with patients’ medical history may have compromised the qualitative evaluation of findings, and, thus, contributed to an underestimation of reliability. On the other hand, this approach could also be viewed as a worst-case-scenario, reflecting the conditions of a busy real-world clinical practice and testing the applicability of FAP-RADS. Another potential limitation was that a small subset of patients (*n* = 9) received [^68^Ga]Ga-FAPI-46, as the pharmacokinetics of [^68^Ga]Ga-FAPI-46 differ from those of [^18^F]AlF-FAPI-74. Further tracer-specific statistics would not provide any meaningful results, as the number of patients in the subset was too small. Hence, future FAP-RADS interreader agreement studies with homogeneous tracer cohorts may provide valuable evidence for further validation of FAP-RADS.

## CONCLUSION

FAP-RADS version 1.0 is an appropriate tool for standardized reporting in clinical studies and regular clinical care, with substantial interreader agreement in most categories. Nevertheless, further studies are needed to test this framework for other tumor entities as well as other FAP ligands.

## DISCLOSURE

Frederik Giesel is an advisor at ABX, Telix Pharmaceuticals, α-Fusion, and SOFIE Biosciences; has a patent application for quinolone-based FAP-targeting agents for imaging and therapy in nuclear medicine; and shares a consultancy group for iTheranostics. Rudolf Werner reports speaker honoraria from Novartis/AAA and PentixaPharm and advisory board work for Novartis/AAA and Bayer. Martin Pomper and Steven Rowe are coinventors on a patent for quinolone-based FAP-targeting agents. Steven Rowe is a consultant for Lantheus, Telix Pharmaceuticals, and Blue Earth Diagnostics Ltd. No other potential conflict of interest relevant to this article was reported.

## Notes

KEY POINTS **QUESTION:** Can FAP-RADS version 1.0, as a standardized reporting system for FAP-targeted imaging, achieve reproducible results across different tumor types and among readers with varying levels of experience? **PERTINENT FINDINGS:** In our cohort study, FAP-RADS version 1.0 achieved excellent internal consistency and an overall high interreader agreement, regardless of reader experience level, especially for parameters important for clinical decision-making (LNM, overall FAP-RADS score, and RPT referral rate) for both UC and LC. **IMPLICATIONS FOR PATIENT CARE:** FAP-RADS version 1.0 framework seems to be an appropriate tool for standardized reporting in clinical studies and regular clinical care. Nevertheless, further studies are needed to test this framework also for other tumor entities due to heterogeneous FAP ligand uptake pattern.

## References

[1] Fitzgerald AA Weiner LM. The role of fibroblast activation protein in health and malignancy. Cancer Metastasis Rev. 2020;39:783–803. 32601975 10.1007/s10555-020-09909-3 PMC7487063

[2] Novruzov E Sheikh GT Mamlins E. Meeting upcoming clinical and diagnostic needs in oncologic imaging: a structured reporting system for fibroblast-activation-protein-targeted imaging—FAP-RADS version 1.0. J Nucl Med. 2025;66:1245–1251. 40473461 10.2967/jnumed.125.269914 PMC12320583

[3] Werner RA Bundschuh RA Bundschuh L. Molecular imaging reporting and data systems (MI-RADS): a generalizable framework for targeted radiotracers with theranostic implications. Ann Nucl Med. 2018;32:512–522. 30109562 10.1007/s12149-018-1291-7 PMC6182628

[4] Evangelista L Filippi L. Structured reporting in prostate cancer: the revolution of quality in nuclear medicine scan interpretation. Eur Radiol. 2024;34:1155–1156. 38123691 10.1007/s00330-023-10507-4

[5] Rowe SP Pienta KJ Pomper MG Gorin MA. Proposal for a structured reporting system for prostate-specific membrane antigen-targeted PET imaging: PSMA-RADS version 1.0. J Nucl Med. 2018;59:479–485. 28887401 10.2967/jnumed.117.195255 PMC6910634

[6] Werner RA Solnes LB Javadi MS. SSTR-RADS version 1.0 as a reporting system for SSTR PET imaging and selection of potential PRRT candidates: a proposed standardization framework. J Nucl Med. 2018;59:1085–1091. 29572257 10.2967/jnumed.117.206631

[7] Loktev A Lindner T Mier W. A tumor-imaging method targeting cancer-associated fibroblasts. J Nucl Med. 2018;59:1423–1429. 29626120 10.2967/jnumed.118.210435 PMC6126438

[8] Lindner T Loktev A Altmann A. Development of quinoline-based theranostic ligands for the targeting of fibroblast activation protein. J Nucl Med. 2018;59:1415–1422. 29626119 10.2967/jnumed.118.210443

[9] Giesel FL Kratochwil C Lindner T. ^68^Ga-FAPI PET/CT: biodistribution and preliminary dosimetry estimate of 2 DOTA-containing FAP-targeting agents in patients with various cancers. J Nucl Med. 2019;60:386–392. 30072500 10.2967/jnumed.118.215913 PMC6424229

[10] Kratochwil C Flechsig P Lindner T. ^68^Ga-FAPI PET/CT: tracer uptake in 28 different kinds of cancer. J Nucl Med. 2019;60:801–805. 30954939 10.2967/jnumed.119.227967 PMC6581228

[11] Liang HX Huang QW He YM. Comparison of the diagnostic accuracy between ^18^F-FAPI-04 PET/CT and ^18^F-FDG PET/CT in the clinical stage IA of lung adenocarcinoma. J Thorac Dis. 2025;17:661–675. 40083505 10.21037/jtd-24-1658 PMC11898335

[12] Novruzov E Dendl K Ndlovu H. Head-to-head intra-individual comparison of [^68^Ga]-FAPI and [^18^F]-FDG PET/CT in patients with bladder cancer. Mol Imaging Biol. 2022;24:651–658. 35349039 10.1007/s11307-022-01715-3 PMC9296390

[13] Mu X Zhu Z Wang Z. Insights into lung cancer diagnosis and clinical management using [^18^F]F-fibroblast activation protein inhibitor (FAPI)-42 positron emission tomography/computed tomography. Ann Nucl Med. 2025;39:576–587. 40053176 10.1007/s12149-025-02032-9

[14] Koo TK Li MY. A guideline of selecting and reporting intraclass correlation coefficients for reliability research. J Chiropr Med. 2016;15:155–163. 27330520 10.1016/j.jcm.2016.02.012 PMC4913118

[15] Witek JA Brooks AF Viglianti BL Scott PJH. Patent spotlight on theranostics targeting fibroblast activation protein for personalized cancer care. Pharm Pat Anal. 2024;13:149–160. 40328490 10.1080/20468954.2025.2500811 PMC12367103

[16] Ruan D Wu S Lin X. Current status of FAP-directed cancer theranostics: a bibliometric analysis. Biophys Rep. 2024;10:388–402. 39758423 10.52601/bpr.2024.240022 PMC11693499

[17] Zboralski D Hoehne A Bredenbeck A. Preclinical evaluation of FAP-2286 for fibroblast activation protein targeted radionuclide imaging and therapy. Eur J Nucl Med Mol Imaging. 2022;49:3651–3667. 35608703 10.1007/s00259-022-05842-5 PMC9399058

[18] Holzgreve A Hellwig D Barthel H. PET imaging utilization and trends in Germany: a comprehensive survey. Eur J Nucl Med Mol Imaging. 2025;52:4390–4398. 40317303 10.1007/s00259-025-07323-x PMC12491347

[19] Baum RP Novruzov E Zhao T. Radiomolecular theranostics with fibroblast-activation-protein inhibitors and peptides. Semin Nucl Med. 2024;54:537–556. 39019653 10.1053/j.semnuclmed.2024.05.010

[20] Demmert TT Pomykala KL Lanzafame H. Oncologic staging with ^68^Ga-FAPI PET/CT demonstrates a lower rate of nonspecific lymph node findings than ^18^F-FDG PET/CT. J Nucl Med. 2023;64:1906–1909. 37734836 10.2967/jnumed.123.265751

[21] Kratochwil C Fendler WP Eiber M. Joint EANM/SNMMI procedure guideline for the use of ^177^Lu-labeled PSMA-targeted radioligand therapy (^177^Lu-PSMA-RLT). Eur J Nucl Med Mol Imaging. 2023;50:2830–2845. 37246997 10.1007/s00259-023-06255-8 PMC10317889

[22] Kayal G Roseland ME Wang C. Multicycle dosimetric behavior and dose-effect relationships in [^177^Lu]Lu-DOTATATE peptide receptor radionuclide therapy. J Nucl Med. 2025;66:900–908. 40274371 10.2967/jnumed.124.269389 PMC12175996

[23] Ulaner GA VanderMolen LA Li G Ferreira D. Dotatate PET/CT and ^225^Ac-Dotatate therapy for somatostatin receptor–expressing metastatic breast cancer. Radiology. 2024;312:e233408. 39078299 10.1148/radiol.233408

